# A249 RETROSPECTIVE CHART REVIEW OF PATIENTS WITH MALNUTRITION REQUIRING ADVANCED NUTRITIONAL SUPPORT POST BARIATRIC SURGERY

**DOI:** 10.1093/jcag/gwab049.248

**Published:** 2022-02-21

**Authors:** L Tsang, M Fredriksen, J Jin, L Gramlich

**Affiliations:** Gastroenterology, University of Alberta Faculty of Medicine and Dentistry, Edmonton, AB, Canada

## Abstract

**Background:**

Obesity is a global pandemic with a steady increase in global BMI since 1975. Bariatric surgery is an effective treatment for patients with severe obesity in order to sustain long-term weight loss and reduce comorbidity burden and mortality associated with obesity. However, post-bariatric surgery patients face nutritional complications ranging from micronutrient deficiencies to intestinal failure requiring total parenteral nutrition (TPN). From our institution of home TPN patients, 6.4% of patients had bariatric surgery. Intestinal failure is a burdensome diagnosis for patients, and there is a paucity of literature characterizing patients post-bariatric surgery who develop intestinal failure.

**Aims:**

We aim to identify the patient characteristics, surgical details, and nutritional traits that predisposed patients to developing intestinal failure requiring advanced nutritional support.

**Methods:**

This is a retrospective chart review of 48 patient admitted to the Royal Alexandra Hospital post-bariatric surgery for nutritional support and followed by the TPN program dieticians and nurses.

**Results:**

Our results show the mean BMI was 49.94 kg/m2 pre-bariatric surgery. Interestingly, the mean BMI at time of hospitalization for bariatric-surgery related complications was 33.210 kg/m2 which is classified as overweight but most of patients were severely malnourished with SGA C (43.8%), SGA B (29.17%), and 8.3% were SGA A. Patients requiring parenteral nutrition post-bariatric surgery are mostly female, developed barriers to oral intake, 15% engaged in medical tourism, 58.3% had an underlying mental health diagnosis, and only 18.8% were on a multivitamin even though it is standard of post-bariatric surgery care. The time between initial bariatric surgery to hospital admission was 11.2 years, and most required ≥1 revisional surgery. The mean age at bariatric surgery was 33.2-years old and the average age at initial hospitalization was 48.9-years old. Patients requiring ≥2 admissions had vertical band gastroplasty or sleeve gastrectomy (both 36.8%) while 21.1% had Roux-en-Y gastric bypass. Of these patients, 39.6% of patients required ≥2 hospital admissions and the mean total days spent in hospital was 57.15 days. While these complications are uncommon, these patients result in multiple prolonged hospitalizations and can be difficult to manage.

**Conclusions:**

Overall, the results of our study will allow the multidisciplinary teams that care for post-bariatric surgery patients to identify patients at risk of intestinal failure and potentially intervene with early enrollment into home nutrition program. With increasing awareness, patients at higher risk can be closely monitored in order to prevent micronutrient deficiencies before they progress to intestinal failure and require lifetime parenteral nutrition.

**Funding Agencies:**

None

